# Helper T cell bias following tuberculosis chemotherapy identifies opportunities for therapeutic vaccination to prevent relapse

**DOI:** 10.1038/s41541-023-00761-4

**Published:** 2023-10-28

**Authors:** Yazmin B. Martinez-Martinez, Matthew B. Huante, Sadhana Chauhan, Kubra F. Naqvi, Preeti Bharaj, Janice J. Endsley

**Affiliations:** 1 Department of Microbiology and Immunology, University of Texas Medical Branch, Galveston, TX 77555 USA; 2 Present Address: Department of Internal Medicine, University of Texas Southwestern Medical Center, Dallas, TX 75390 USA; 3 Present Address: Department of Medicine, Rutgers New Jersey Medical School, Newark, NJ 07103 USA

**Keywords:** Tuberculosis, Lymphocyte activation

## Abstract

Therapeutic vaccines have promise as adjunctive treatment for tuberculosis (TB) or as preventives against TB relapse. An important development challenge is the limited understanding of T helper (Th) cell roles during these stages of disease. A murine model of TB relapse was used to identify changes in Th populations and cytokine microenvironment. Active TB promoted expansion of Th1, Th2, Th17, and Th22 cells and cytokines in the lung. Following drug therapy, pulmonary Th17 and Th22 cells contracted, Th1 cells remained elevated, while Th cells producing IL-4 or IL-10 expanded. At relapse, Th22 cells failed to re-expand in the lung despite a moderate re-expansion of Th1 and Th17 cells and an increase in Th cytokine polyfunctionality. The dynamics of Th populations further differed by tissue compartment and disease presentation. These outcomes identify immune bias by Th subpopulations during TB relapse as candidate mechanisms for pathogenesis and targets for therapeutic vaccination.

## Introduction

As the incidence of COVID-19 declines, tuberculosis (TB) is re-emerging as the leading cause of death due to infection. Annually, 1.6 million deaths and 10.6 million cases of active TB occur due to infection with *Mycobacterium tuberculosis* (Mtb)^1^. With timely diagnosis and compliance to the standard treatment regimen, TB treatment outcomes are usually positive. An important challenge to disease control is development of TB recurrence that is associated with reduced treatment success, acquired drug resistance, higher mortality rate, and other poor clinical outcomes^2^. Risk factors for TB recurrence include human immunodeficiency virus (HIV), diabetes mellitus, smoking, air pollution, previous multi-drug resistance, and underserved population status, such as those that are incarcerated, homeless, or suffering from drug addiction^3–9^. Of those with apparent clinical cure following TB treatment, TB recurrence will occur in an estimated 7% (700,000) of all, and up to 24.4% of persons with HIV co-infection^1,10^. TB recurrence occurs due to reinfection with a new Mtb strain, or regrowth of the original Mtb strain following a drug treatment that fails to eradicate viable organisms. The latter outcome, known as relapse, occurs when patients fail to clear Mtb infection, mainly due to inadequate drug treatment, unrecognized drug resistance, immune compromise, or incorrect adherence to the therapy^9^. Relapse is the most common cause of recurrence in low TB burden countries, while reinfection plays a greater role in high TB burden countries^2,10^. Whole genome sequencing results demonstrate that recurrence within one year of treatment primarily results from relapse rather than re-infection^11–16^, with relapse contributing to 70% of recurrences, more than previously appreciated^17^. Development of an effective therapeutic vaccine as adjunctive treatment or preventive measure to reduce TB relapse is thus a priority.

Residual lung cavitation observed by X-ray, and positive sputum culture observed 2 months after initiation of anti-tubercular therapy, are good prognostic indicators of relapse risk^10,18^. Recent observations by PET-CT, however, demonstrate metabolically active TB lesions associated with ongoing Mtb transcription (mRNA) in lung of those with apparent clinical cure following standard TB therapy^19^. Follow up imaging revealed that lesions in most subjects resolved over time, consistent with clinical outcomes^19^. These findings challenge the paradigm that most patients, including those without known risk factors for relapse, reach sterilization after therapy. The overlap in areas of observed lung pathology following infection and post-drug treatment relapse also suggest Mtb regrowth may occur in areas of incomplete sterilization^20^. Several lesions arose de novo as well, suggesting regrowth from disseminated mycobacteria^20^. Assessment of human radiological images further revealed that 90% of cavity lesions were in the apical segments of the lung, consistent with recurrent TB cavity localization^21^. These results suggest that Mtb persistence is underestimated following drug treatment and that host immune status is an important determinant of the clinical outcome.

Development of vaccines or therapies to prevent relapse are limited by gaps in knowledge relating to mechanisms of protective immunity and correlates of pulmonary clearance. The essential role for cell mediated immunity in mycobacterial containment is well described. T cells, and especially (CD3+ CD4+) T helper (Th) cells are required for immunity to Mtb^22^. Generation of antigen-specific Th populations by infection or vaccination corresponds with protective outcomes^22–24^. The immunology of relapse, however, is poorly understood due to a paucity of animal models, challenges in obtaining human samples, and lack of investigation^25^. Limited studies of human biomarkers suggest that elevated plasma IL-6, IL-1β, and soluble IL-1Ra are associated with greater risk for recurrence^26^. Assessment of recurrent TB in HIV co-infected patients identified an association of IL-1β with both protection and increased risk, depending on the innate immune cellular sources^27^. Plasma cytokine biosignatures described in a small number of human studies suggest immune suppression or dysfunction at relapse^28^. In a standard mouse model of TB relapse, reduced plasma cytokines (IL-17, IFN-γ, IL-6, TNF, CXCL9, and CXCL10) and increased pulmonary IFN-γ+ lymphocytes were observed during regrowth compared to initial infection^29^. In a humanized mouse model of TB relapse, HIV infection promoted relapse associated with suppression of pulmonary IL-17^30^. Combined with the established risk factors for relapse, these limited experiments suggest important differences in T cell and other immune responses during primary and recrudescent stages of TB. Assessments of human lung compartments are especially inadequate due to sample constraints.

We applied an experimental model of TB relapse in a standard C57BL/6 mouse to determine changes in Th populations and polyfunctional activity in the blood, lung, and spleen across active TB (ATB), post-drug TB (PDTB), and TB relapse. A pro-inflammatory cytokine signature and increases in Th1, Th17, Th1Th17, Th22, and Th2 cells were observed in lung during ATB. This response was markedly subdued after antibiotic treatment and characterized by an increase of Th2 and Th IL-10+ cells, irrespective of infection status. The effector cytokine response observed during ATB was not mirrored at relapse TB, and pulmonary Th populations were characterized by moderate increases in Th1 and Th17 cells, an increase in cytokine polyfunctionality, and a notable lack of Th22. Overall, our results demonstrate a marked difference in the predominance of pulmonary Th subpopulations at TB relapse, as compared to ATB and to other tissue compartments. These findings represent an important advance in our understanding of the host immune status during the post-drug and relapse stage needed to guide development of therapeutic vaccine and host-directed therapy approaches.

## Results

### Regrowth of paucibacillary Mtb following drug treatment in a murine model of TB relapse

**Fig. 1:**
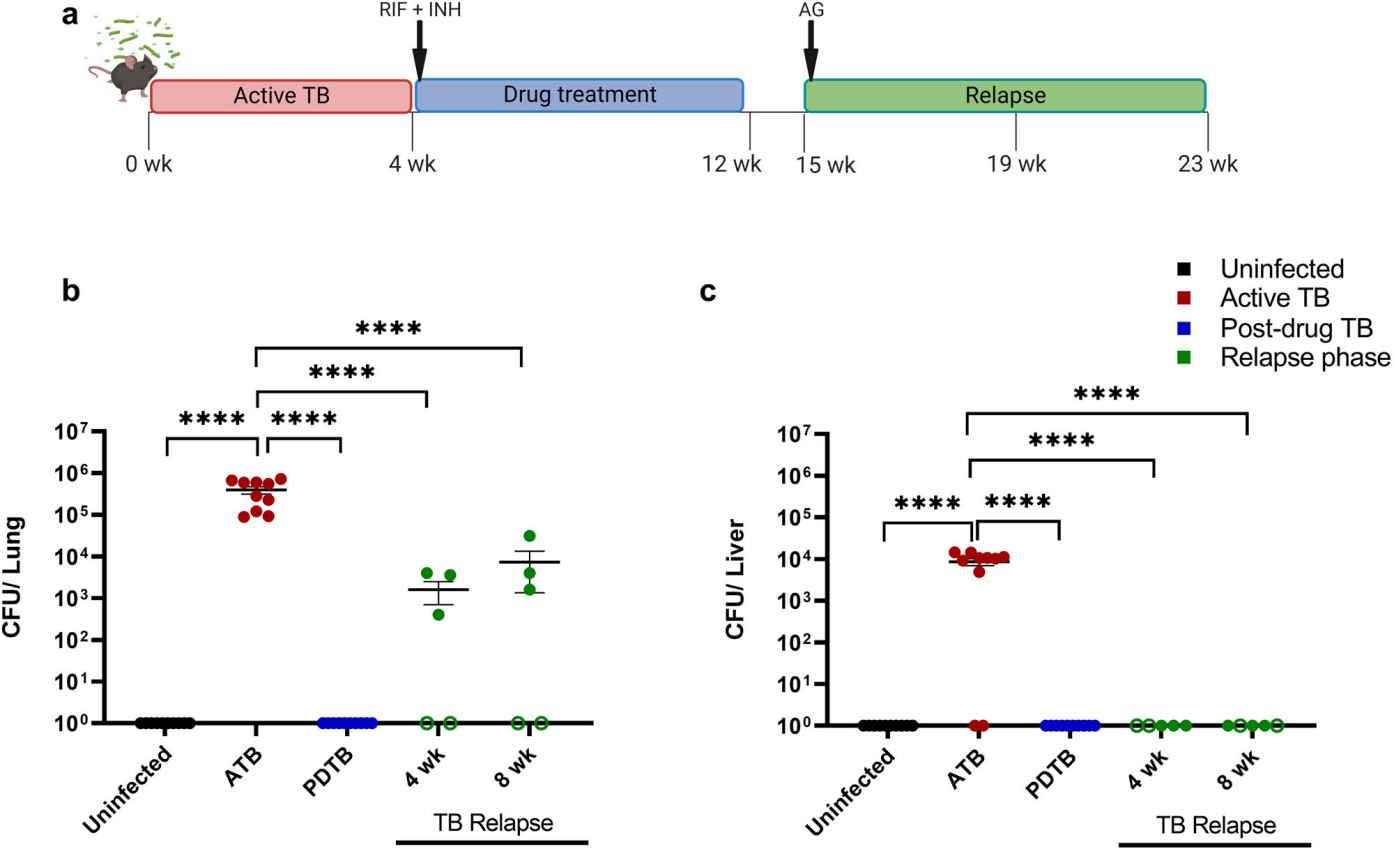
Regrowth of paucibacillary Mtb following drug treatment in a murine model of TB relapse. **a** Mouse model of TB relapse after drug treatment. C57BL/6J were infected via aerosol with 100 CFU of Mtb HN878. At day 28, an 8-week course of antibiotic treatment was given using an oral regimen of 0.1 mg/mL each of Rifampicin (RIF) and Isoniazid (INH), provided in drinking water, until day 81. After three weeks of no treatment (day 105 or week 15) the relapse phase was initiated by treatment with *ad libitum* water containing 2.5% aminoguanidine (AG) supplemented with 10% glucose to increase palatability, until day 161. Mice were humanely euthanized at the end of each experimental phase, corresponding to weeks 4, 12, 19, and 23. Created with BioRender.com. **b**, **c** Bacterial burden in lung (**b**) and liver (**c**), across ATB (*n* = 10), post-drug treatment reaching paucibacillary stage (*n* = 10), and relapse phase at 4 (*n* = 5) and 8 weeks (*n* = 5) of TB relapse, compared to uninfected (*n* = 10). Open green symbols indicate animals that did not relapse. Comparisons across treatment stages were analyzed using one-way ANOVA followed by Tukey test for multiple comparisons. Data shown as mean ± SEM. ****p < 0.0001.

To identify the Th populations that participate in immune responses to post-drug Mtb relapse, we employed a murine model of drug-induced paucibacillary TB similar to that described by Botha and Ryffel^31^. As shown in the experiment schematic in Fig. 1a, C57BL/6J mice infected with 100 colony forming units (CFU) via an aerosol route developed ATB by 4 weeks p.i. as evidenced by a mycobacterial burden of approximately 10^6^ CFU/lung (Fig. 1b). Following 8 weeks of treatment with RIF and INH, Mtb CFU in the lung were below the limit of detection (Fig. 1b) in the PDTB stage.

Three weeks following the end of drug treatment, relapse was induced using AG as described^31,32^. Inhibition of NOS2 by AG is used in murine models of relapse as it impairs antibacterial activity of macrophages which is a common defect associated with diseases that increase relapse risk. Relapse is significantly delayed and variable among animals in the absence of interventions such as AG and thus presents significant challenges for assessment of immune endpoints that occur at relapse^32^. Consistent with these previous observations, a protracted rate of relapse was observed in our model in the absence of AG. As shown in Supplementary Fig. 1a, only 33% of animals presented Mtb regrowth at 11 weeks (or experimental week 23) following cessation of drug treatment. This result was observed despite development of similar pulmonary burden at ATB and PDTB stages to experiments that utilized AG (Supplementary Fig. 1a, b).

Following AG administration, 60% of mice developed relapse at 4- or 8-weeks post AG (Fig. 1b), with no significant difference in bacterial burden observed to occur between both endpoints. The pulmonary burden in animals that relapsed was lower than that observed at 4 weeks post-infection (ATB), 10^3^–10^4^ and 10^6^ CFU/lung lobe, respectively (Fig. 1b). These differences in bacterial burden across active TB, drug treatment, and different relapse timepoints observed in the model provided relevant baseline endpoints to determine different Th cell profiles corresponding to disease stage. To assess bacterial dissemination beyond the lung, and preserve the spleen for immune response assessment, we measured the liver CFU in the ATB, PDTB, and relapse phases. In contrast to lung, we observed that only 80% of the mice displayed dissemination during ATB while CFU were below the limit of detection at both the 4- and 8-weeks relapse timepoints (Fig. 1c).

### Pulmonary Th cell immune profile discriminates between active and relapse TB

**Fig. 2:**
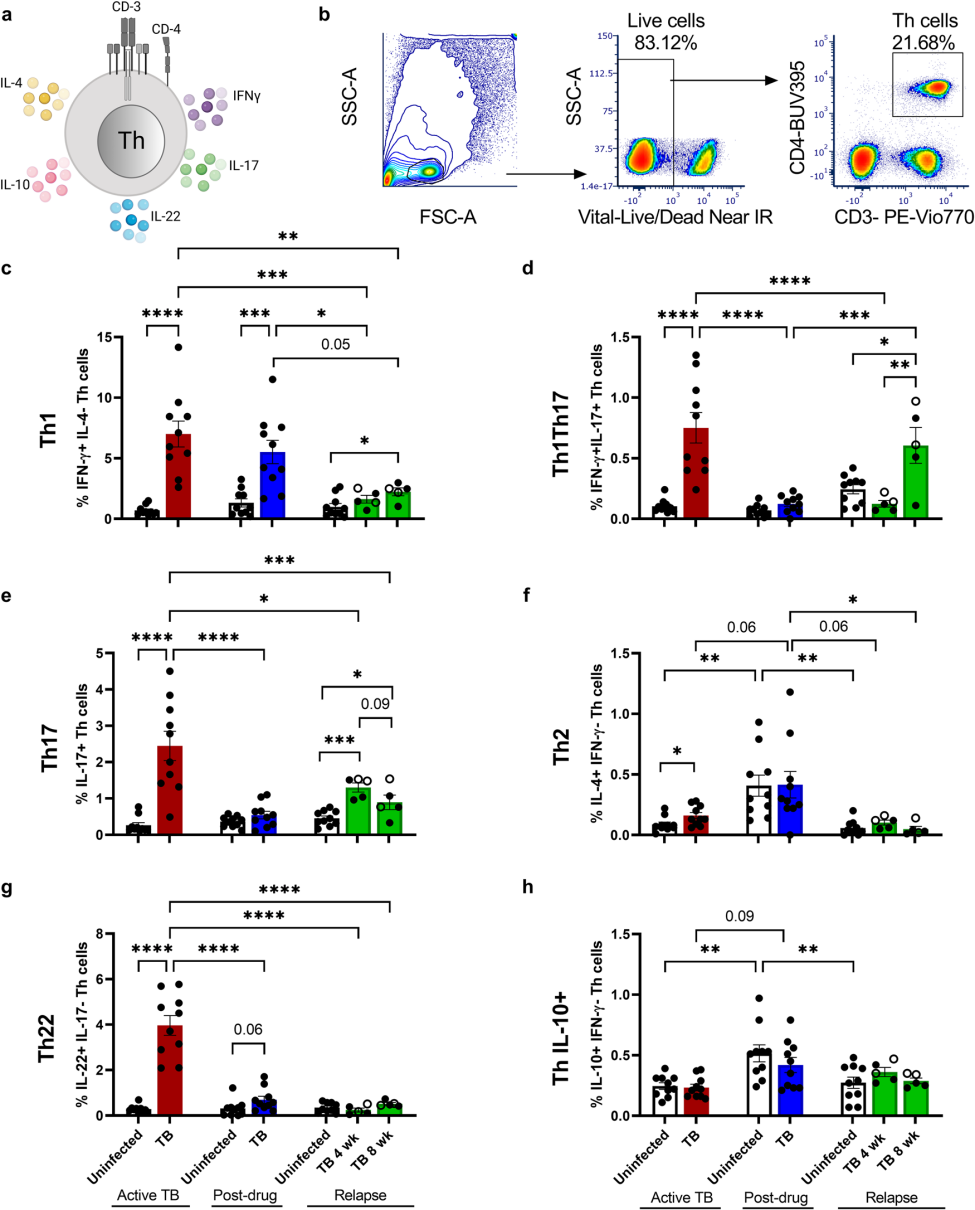
Pulmonary Th cell immune profile discriminates between active and relapse TB. **a** Schematic figure for the different markers used in flow cytometry for these experiments, created with BioRender.com. **b** Gating strategy utilized for Th cell analysis. **c**–**h** Percentage of specific-cytokine producers among Th cell subsets (CD3 + CD4+ lymphocytes) in lung after active TB (red, *n* = 10), post-drug treatment (blue, *n* = 10), and 4 and 8 weeks relapse (green, *n* = 5/group), compared to the uninfected controls (white, *n* = 10/group). **c** Th1 (IFN-γ + IL-4-) cells. **d** Th1Th17 cells that are double positive for IFN-γ and IL-17. **e** Th17 (IL-17 + ) cells. **f** Th2 (IL-4 + IFN-γ-) cells. **g** Th22 (IL-22 + IL-17-) cells. **h** Th cell percentages producing IL-10 (IL-10 + IFN-γ-). Open green symbols indicate animals that did not relapse. A Student’s t-test was used to determine differences between two groups within treatment phase. Comparisons in relapse, or across treatment stages were analyzed using one-way ANOVA followed by Tukey test for multiple comparisons. Data shown as mean ± SEM. *p < 0.05, **p < 0.01, ***p < 0.001, ****p < 0.0001.

The prevalence of several Th subtypes have been recently described in human lung and blood following Mtb infection^33,34^. The dynamics of Th populations responses at PDTB and TB relapse in the lung, however, remain to be elucidated. To understand the cellular immune responses that promote immune containment following drug therapy, we analyzed the Th population changes in our relapse mouse model by flow cytometry (Fig. 2a, b, Supplementary Fig. 2). Use of non-specific stimuli in an ex vivo approach allowed an assessment of the Th population dynamics driven by proliferating or persisting Mtb in vivo that was not limited by T cell receptor epitope recognition. During ATB, the total number and % of the Th (CD3 + CD4 +) pool in the lung was elevated (Supplementary Fig. 3b, d), in the absence of a significant increase in total lymphocytes (Supplementary Fig. 3a, c). The number and percentage of both lymphocytes and Th cells increased at PDTB in comparison to the uninfected controls, although Th cell numbers were reduced compared to ATB (Supplementary Fig. 3a–d). During relapse, the number of both total and Th lymphocytes increased in the absence of changes in percentages relative to controls (Supplementary Fig. 3). Th cells accounted for approximately 10% of the lymphocytes at relapse, compared to 40% in ATB. These results suggest greater increases in non-Th lymphocyte populations in relapse compared to ATB or PDTB. Notably, a decline in Th cells between 4 and 8 weeks of relapse was also observed (Supplementary Fig. 3b, d).

A breakdown of pulmonary Th subpopulations demonstrates that the increase in Th cells during ATB was due to Th1 (IFN-γ + IL-4-), Th17 (IL-17+), Th22 (IL-22 + IL-17-) as well as Th1Th17 (IFN-γ + IL-17 +) populations (Fig. 2c–e, g). After drug-treatment, the Th17, Th22, and Th1Th17 populations contracted (Fig. 2d, e, g), in association with a decline in total Th cells (Supplementary Fig. 3b, d) and pulmonary Mtb burden (Fig. 1b). In contrast, Th1 cells remain elevated (Fig. 2c) at the PDTB endpoint despite a lack of detectable CFU (Fig. 1b, c). Th1 cells displayed a moderate increase at 4 and 8 weeks of relapse that reached significance compared to uninfected controls at 8 weeks (Fig. 2c). The response was similar between 4 and 8 weeks of relapse and the overall response was reduced compared to both ATB and PDTB. In contrast, Th17 cells increased significantly compared to uninfected controls following 4 weeks of relapse, and trended toward decrease between 4 to 8 weeks (Fig. 2e). The multifunctional Th1Th17 cells (described as Th17.1, Th*, or pathogenic Th17)^35–37^ displayed a pattern similar to Th17 at ATB and PDTB, and increased similar to Th1 at 8 weeks of relapse (Fig. 2c, d). Th22 cells presented a very different response at relapse compared to ATB and other Th populations. During ATB, Th22 were abundant among the Th population in the lung. At relapse, Th22 cells were a minimal population of Th cells that failed to expand, compared to uninfected controls, in response to increasing bacterial burden at both 4 and 8 weeks (Fig. 2g). Assessment of relationships between Th subset responses and bacterial burden in lung revealed a lack of significant correlation during ATB and PDTB due to limited variability in CFU among animals within infection groups. A positive relationship (r = 0.7, *p* = 0.009) between the percentage of Th1 cells and lung CFU was observed at relapse, while Th17 cells failed to similarly correlate due to the trend toward decline at 8 weeks.

We further determined changes in the Th2 (CD3 + CD4 + IL-4 + IFN-γ-) and Th IL-10+ (CD3 + CD4 + IL-10 + IFN-γ-) populations across disease stages. Th2 cells were also elevated during ATB (Fig. 2f). Interestingly, both Th2 and Th IL-10+ cells were increased after drug administration in lung, in both the uninfected and Mtb-infected groups. These results suggest immune regulatory responses due to TB chemotherapy that occur irrespective of infection status (Fig. 2f, h). In contrast to the other Th1 and Th17 populations, both Th2 and Th IL-10+ returned to basal levels during TB relapse similar to Th22. Within the 4 and 8 week relapse groups, there were similar responses observed in animals that had detectable relapse compared to those that did not although results from non-relapsing animals were often at the higher end of the group range. In control experiments, AG use demonstrated no biologically significant effect on the baseline Th subsets in lung of uninfected animals after 4 and 8 weeks of administration (Supplementary Fig. 1b, c lung).

### Cytokine signatures demonstrate reduced immune response in lung during TB relapse

**Fig. 3:**
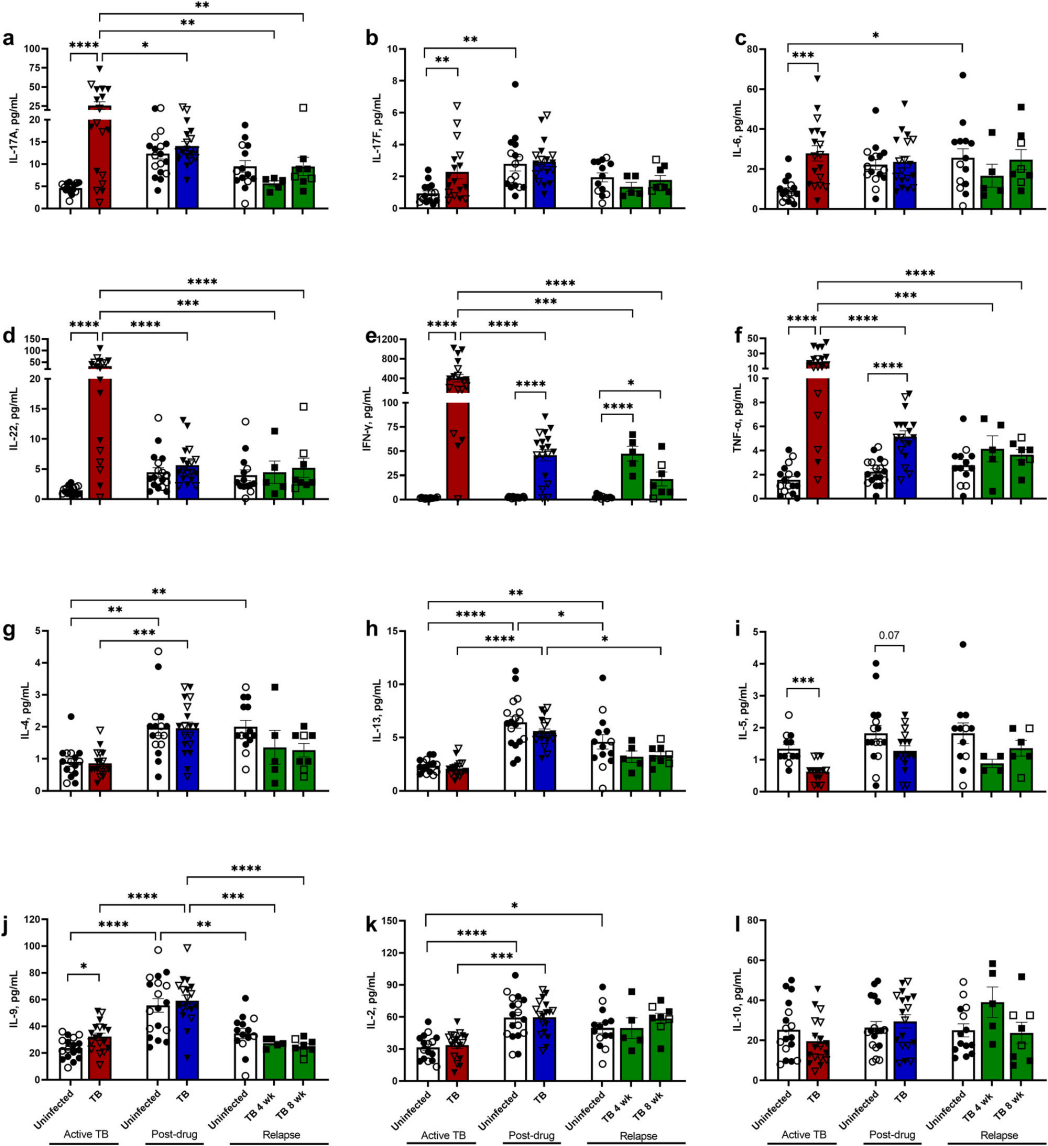
Cytokine signatures demonstrate reduced immune response in lung during TB relapse. Right lung (superior, middle, inferior and post-caval lobes) was collected in 1 mL of sterile PBS, homogenized and centrifuged. Supernatants were collected and stored at −80 °C for subsequent analysis. Lung cytokines are depicted in pg/mL across active TB (red, *n* = 18), post-drug treatment (blue, *n* = 18), and 4 and 8 weeks relapse (green, *n* = 5 and 8, respectively), in comparison to their controls (white, *n* = 16, 18 and 14, respectively). Open and closed symbols indicate data from 2 independent experiments. **a**–**c** Th17-related lung cytokine production for IL-17A (**a**), IL-17F (**b**), and IL-6 (**c**) across infection phases. **d**–**f** Lung pro-inflammatory cytokines for Th22 and Th1 producers: IL-22 (**d**), IFN-γ (**e**), and TNF (**f**) in pg/mL for comparison to their respective Th producers. **g**–**l** Lung anti-inflammatory cytokines production across experimental phases in pg/mL: IL-4 (**g**), IL-13 (**h**), IL-5 (**i**), IL-9 (**j**), IL-2 (**k**), and IL-10 (**l**). A Student’s t-test was used to determine differences between two groups within treatment phase. Comparisons in relapse, or across treatment stages were analyzed using one-way ANOVA followed by Tukey test for multiple comparisons. Data shown as mean ± SEM. *p < 0.05, **p < 0.01, ***p < 0.001, ****p < 0.0001.

To determine how the Th cell profile found at ATB, PDTB, and relapse phases alters the effector cytokine profile, we used a cytokine bead array approach. Our observation that pro-inflammatory Th (Th1, Th17, Th1Th17) and Th22 populations predominate during ATB was further supported by the increased IFN-γ, TNF, IL-17A, IL-17F, IL-6, and IL-22 cytokines observed in lung supernatants (Fig. 3a–f, red). Levels of Th2-derived and anti-inflammatory cytokines generally reflected the Th subpopulation bias at ATB and other stages. Increased IL-9 was observed during ATB while levels of IL-10, IL-4, IL-13, IL-2, and IL-5 were similar or decreased compared to uninfected (Fig. 3g–l).

Consistent with the observed reduction in bacterial burden, most pro-inflammatory cytokine responses returned to levels comparable to uninfected controls following drug treatment, except for IFN-γ and TNF which remained elevated. In contrast, greater production of IL-4, IL-13, IL-9, and IL-2 was observed following drug treatment irrespective of infection status (Fig. 3g, h, j, k), consistent with the observation of increased Th2 populations (Fig. 2f) at the PDTB stage. Surprisingly, pulmonary cytokine responses were generally limited during relapse, compared to ATB, despite the regrowth of Mtb in the lung. An important note is that mycobacterial burden was lower, even at 8 weeks of relapse, compared to 4 weeks ATB. A pattern of cytokine activation proportional to CFU increase at relapse, however, was not observed. The only pro-inflammatory cytokine that was significantly increased at relapse was IFN-γ; thus, IFN-γ was the only cytokine activated by infection in all three disease presentations. Levels of IL-17A, IL-17F, IL-22, TNF, and IL-6 remained similar to control (Fig. 3a–f in green). A moderate increase in IL-10, which did not reach significance, was also observed at 4 weeks of relapse.

### Polyfunctionality of lung Th cells changes with disease progression

**Fig. 4:**
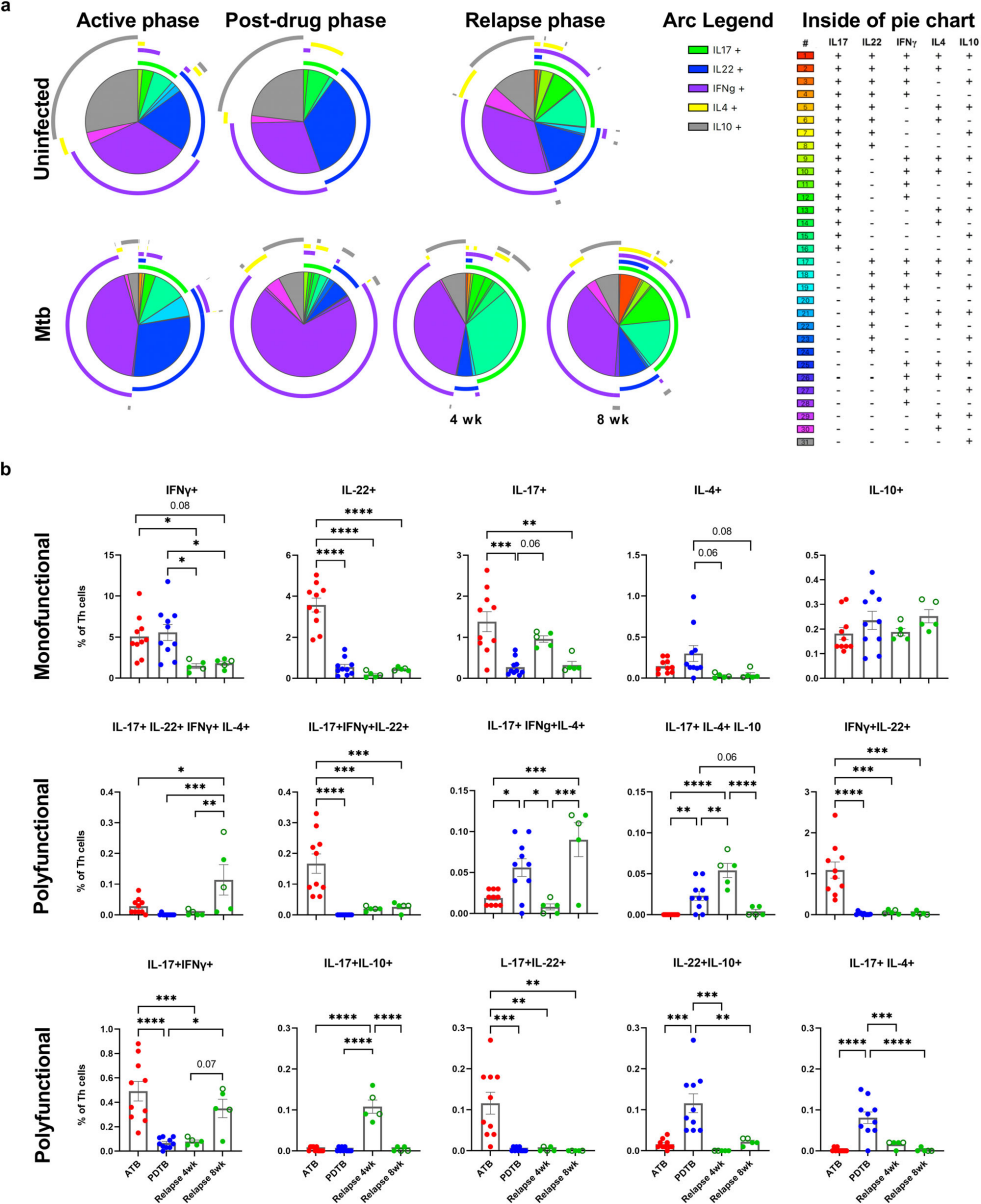
Polyfunctionality of lung Th cells changes with disease progression. Polyfunctional analysis of cytokine producing CD3 + CD4+ cells, determined by flow cytometry and post-acquisition Boolean gating, across active (*n* = 10), post-drug (*n* = 10), and relapse TB phases (4 and 8 weeks, *n* = 5/ group), by Pestle and SPICE. **a** Top panels represent the uninfected groups at ATB, PDTB, and relapse 8 weeks (*n* = 10/ group). Bottom panels represent Mtb-infected groups of each of the phases. Outside of the pie charts, the five arc colors are depicted in the arc legends showing overlap for one or more cytokines. The inside pie color legends (31 colors) are depicted at the right for each subgroup, showing positive or negative production of each of the combination for the five cytokines. **b** The fifteen populations demonstrating the most significant differences in Mtb-infected groups across disease stages are shown. Monofunctional populations are shown in the top panels while polyfunctional populations are shown in the middle and bottom panels. Open green symbols indicate animals that did not relapse. Comparisons across treatment stages were analyzed using one-way ANOVA followed by Tukey test for multiple comparisons. Data shown as mean ± SEM. *p < 0.05, **p < 0.01, ***p < 0.001, ****p < 0.0001.

The observed differences in the Th1Th17 population across the stages of infection in our study suggested the potential for other differences in polyfunctionality of the lung response. To develop more in-depth profiles of polyfunctionality, Boolean analysis of IL-17, IL-22, IFN-γ, IL-4, and IL-10 flow cytometry data was performed by FCS Express and assessed using PESTLE and SPICE (Fig. 4). Consistent with other studies^38^ the majority of Th cells (87.6 to 98.5%) in the lung were characterized as inactive (i.e., not producing any of those 5 cytokines) (Supplementary Fig. 4a). These inactive Th cells are most abundant during TB relapse (96.8%), compared to ATB and PDTB phases (87.6%, and 92.5% inactive, respectively) (Supplementary Fig. 4a, b).

Th cells producing a mono-cytokine profile was the most common observation across all treatments, and the uninfected control groups (Fig. 4a, b). Highly functional populations, those rare cells producing 4 or 5 cytokines simultaneously, were generally limited across all treatment groups. A relative increase in cells defined by an IL-17 + IL-22 + IFN-γ + IL4 + polyfunctional profile was observed at 8 weeks of relapse. Across infection phases, Th cells with an IFN-γ+ mono-cytokine profile (purple arc legend) were the predominant group. Th cells producing either IL-22 or IFN-γ represented approximately 75% of activated Th cells during ATB, and a significant population of IFN-γ + IL-22+ polyfunctional cells were also observed (Fig. 4b). As infection progressed from ATB to relapse, Th cells producing IL-22 decreased (blue arc legend), while polyfunctional groups that include IL-17 increased (green arc legend groups) (Fig. 4a, b).

IL-17 was the most frequent contributor to polyfunctional cytokine profiles compared to other effector cytokines. During ATB, among these IL-17+ cytokine groups, the majority were mono-cytokine producers followed by polyfunctional cells that co-produced IL-17 along with IFN-γ+ and/ or IL-22+ (Fig. 4a, b). During PDTB, the Th profile was predominantly mono-cytokine and polyfunctional, with populations of IL-4+ or IL-10+ cells most abundant (Fig. 4b), consistent with their increase during PDTB irrespective of infection (Fig. 2f, h). Of note, increased populations of IL-22 + IL-10+ or IL-17 + IL-4+ Th cells were uniquely observed at this stage (Fig. 4b). IL-17 polyfunctionality was greatest during relapse and Th cells expressing IL-17 are the only polyfunctional populations which re-expand at relapse following a contraction in the PDTB stage. Comparison of the “pathogenic” Th1Th17 and the “protective” Th17 (Th17IL-10 +), identified the 4-week relapse as the only timepoint when Th17IL-10+ populations were observed (Fig. 4b), which could relate to the moderate IL-10 cytokine increase at that stage (Fig. 3l). In lung of uninfected mice, Th cells actively producing cytokines were limited (Supplementary Fig. 4a, b) and characterized primarily by mono-cytokine production of IL-10, IFN-γ, or IL-22 (Fig. 4a).

### Th populations increase in blood during ATB, and in spleen at TB relapse

**Fig. 5:**
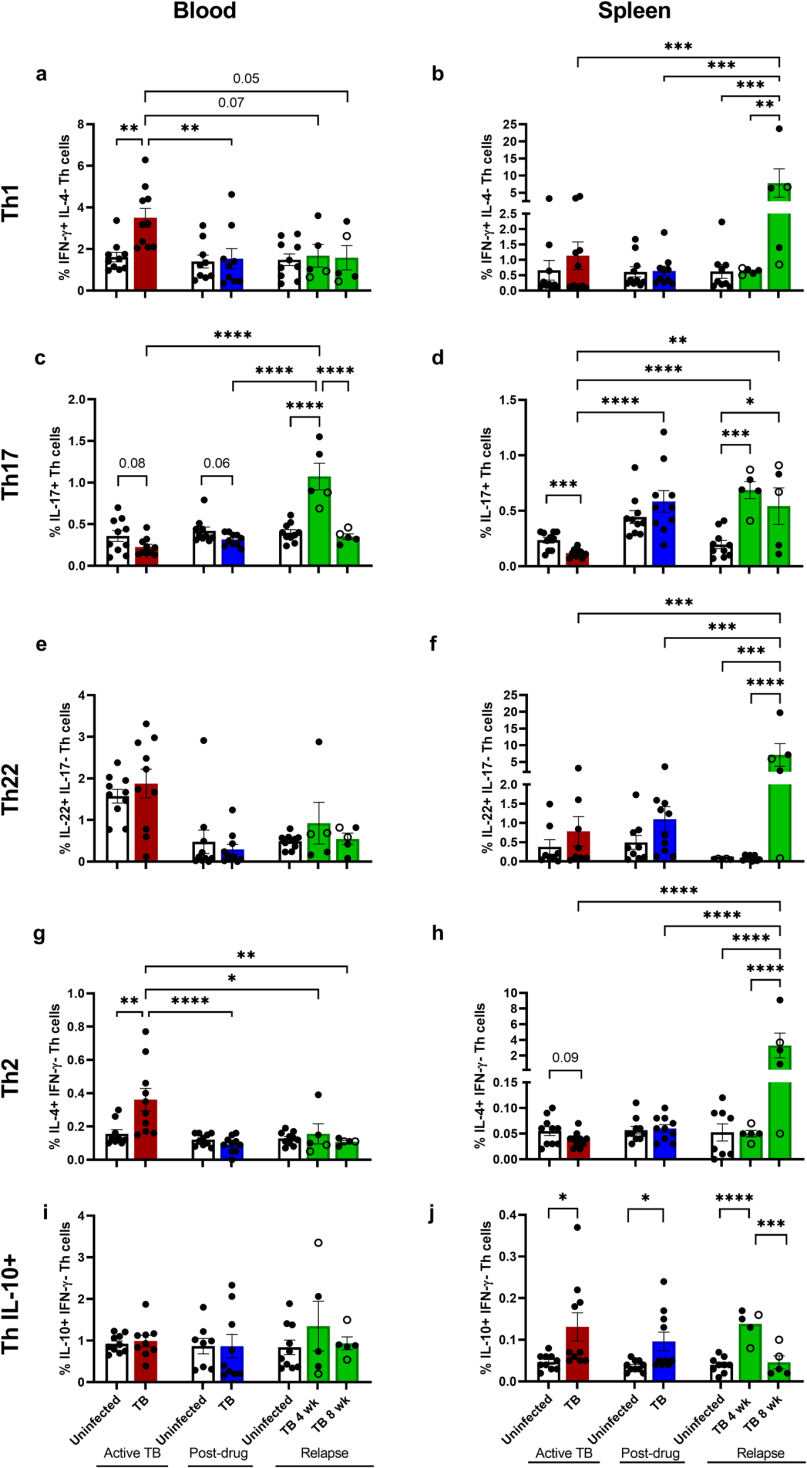
Th populations increase in blood during ATB and in spleen at TB relapse. Subpopulations of Th cells are depicted as percentage of specific-cytokine producers among CD3 + CD4+ lymphocytes for blood (**a**, **c**, **e**, **g**, **i**) and spleen (**b**, **d**, **f**, **h**, **j**) after 4 weeks of Mtb infection (red, *n* = 10), post-drug treatment (blue, *n* = 10), and 4 and 8 weeks relapse (green, *n* = 5/group), compared to the uninfected controls (white, *n* = 10/group). **a**, **b** Th1 (IFN-γ + IL-4-CD3 + CD4 +) cells at different TB infection phases in blood (**a**), and spleen (**b**). **c**, **d** Th17 (IL-17 + CD3 + CD4 +) cells in blood (**c**), and in spleen (**d**). **e**, **f** Th22 (IL-22 + IL-17-CD3 + CD4+) cells in blood (**e**), and spleen (**f**). **g**, **h** Th2 (IL-4 + IFN-γ-CD3 + CD4+) cell percentages among Th cells (CD3 + CD4+) in blood (**g**), and spleen (**h**). **i**, **j** Th cells producing IL-10 (IL-10 + IFN-γ-CD3 + CD4+) in blood (**i**), and spleen (**j**). Open green symbols indicate animals that did not relapse. A Student’s t-test was used to determine differences between two groups within treatment phase. Comparisons in relapse and across treatment stages were analyzed using one-way ANOVA followed by Tukey test for multiple comparisons. Data shown as mean ± SEM. ns: not significant, *p < 0.05, **p < 0.01, ***p < 0.001, ****p < 0.0001.

To investigate if the peripheral immune system reflects the lung microenvironment across infection and treatment in TB, we performed flow cytometric analysis of cells from the blood and spleen. Similar to the lung (Fig. 2c, f), Th1 and Th2 populations increased in the blood during ATB (Fig. 5a, g). In contrast to the lung, the Th22 population in the blood failed to expand during ATB (Fig. 5e). The Th17 population in blood displayed a decreased trend during ATB (*p* = 0.08), and at PDTB (*p* = 0.06) also in contrast to the lung response. A marked expansion of Th17 cells was observed at 4, and declined by 8 weeks of relapse (Fig. 5c). The percent of other Th populations in the blood were similar to uninfected controls at relapse regardless of changes during ATB. The expansion of Th2 and Th IL-10+ observed in the lung due to antibiotic treatment was absent in the blood (Fig. 5g, i). An interesting observation in blood, compared to lung, was expansion of the Th1Th17 population during PDTB irrespective of infection (Supplementary Fig. 4d).

Splenic Th populations also differed from both the lung and blood. Th1, Th22, and Th2 populations in spleen remained unchanged compared to uninfected controls during ATB as well as PDTB (Fig. 5b, f, h). In striking contrast to the pulmonary response, Th17 and Th1Th17 populations declined in the spleen during ATB (Fig. 5d, Supplementary Fig. 4c). Recovery of the Th17 response at PDTB was suggested by an increase above levels observed during ATB. At relapse, splenic Th17 cells significantly increased by 4 weeks (Fig. 5d), similar to blood and lung responses at this stage. Marked increases in Th1, Th22, and Th2 were also observed in spleen by 8 weeks of relapse (Fig. 5b, f, h). These observations suggest potential for the spleen as a source of activated Th populations during the TB relapse phase. In contrast to other Th cells in spleen, the Th IL10+ subpopulation was increased compared to controls throughout all three stages of infection, contracting at 8 weeks of relapse (Fig. 5j), although the overall % of Th IL-10+ cells was modest.

**Fig. 6:**
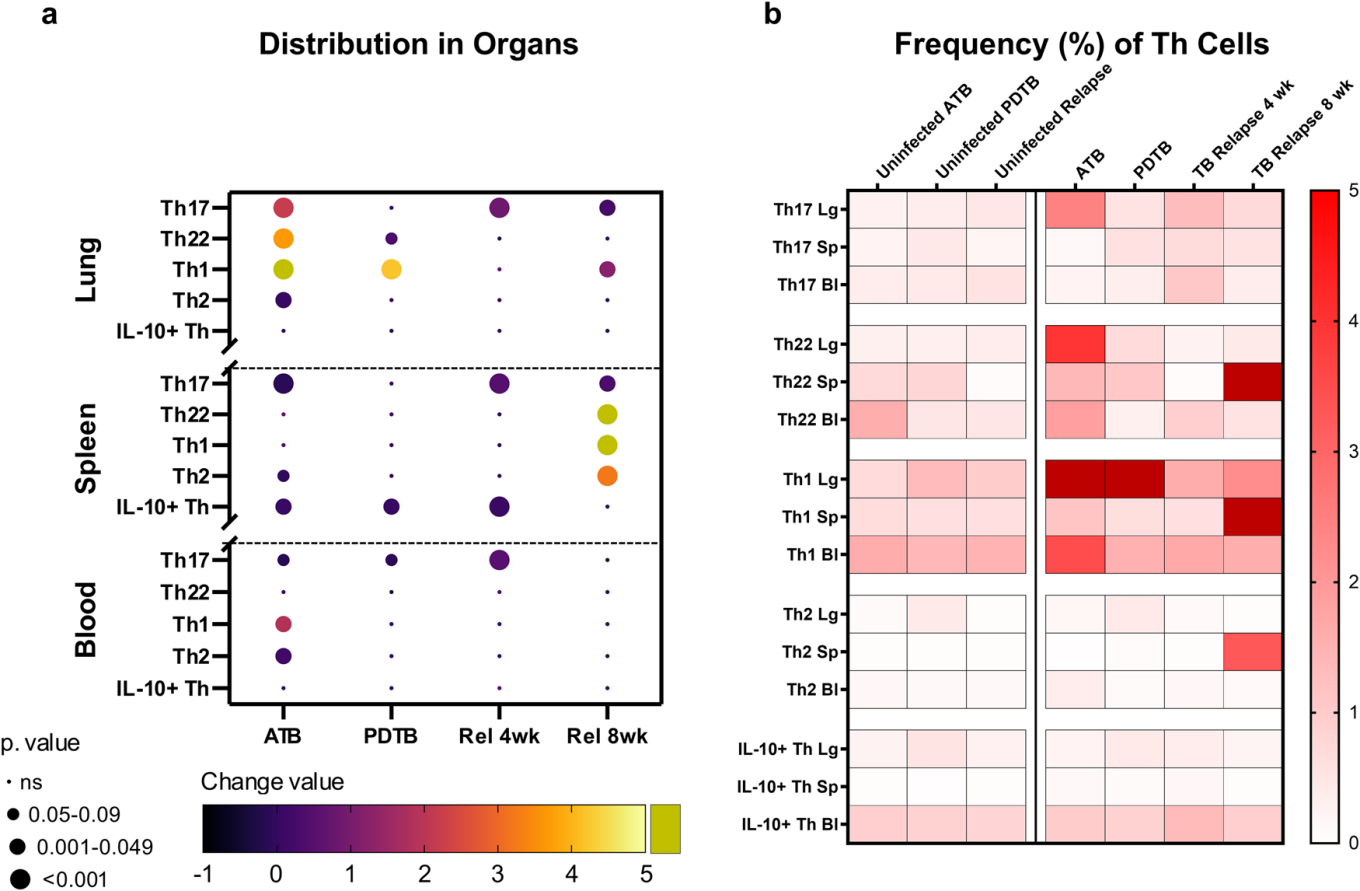
Th cell dynamics of lung, spleen, and blood corresponding with disease presentation. **a** Summary bubble plot comparing the percentage change of each T cell subset in different organs versus its uninfected control. Changes were assessed subtracting the uninfected from the corresponding mean percentage of each phase, to visually assess increasing or decreasing populations due to infection. **b** Heat map comparing the observed cell percentages for each stage and organ. Variation is depicted across percentages of Th17, Th22, Th1, Th2, and Th IL-10+ cells among CD3 + CD4+ cells, in lung (Lg), spleen (Sp), or blood (Bl). Changes were assessed after 4 weeks of infection, post-drug administration, and at 4 and 8 weeks of TB relapse (Rel), for both uninfected (left) and Mtb-infected groups (right).

**Fig. 7:**
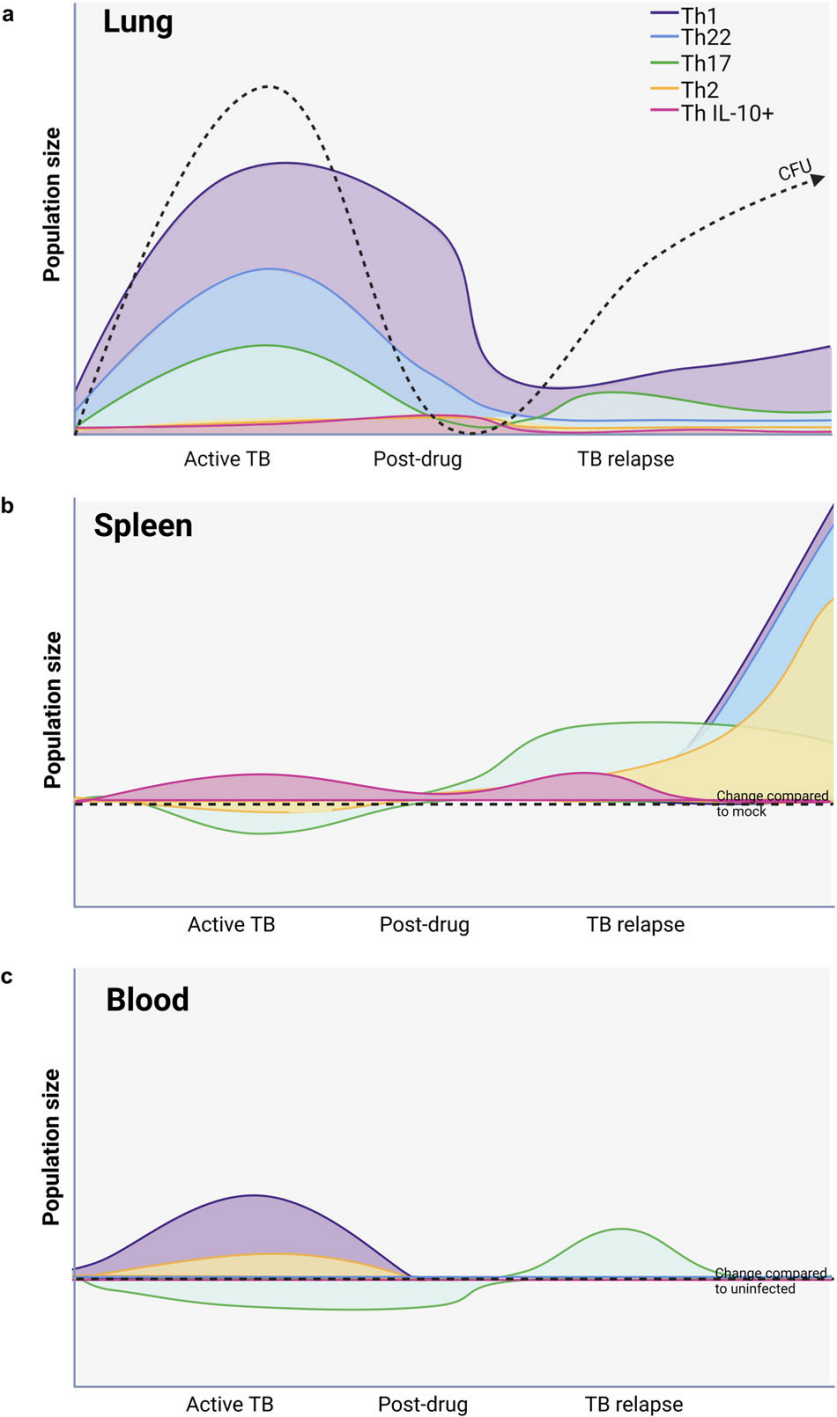
Graphical summary of Th bias following primary infection, after drug treatment, and in relapse TB. Population percentage changes compared to CFU variation in the different infection stages in the lung (**a**), spleen (**b**), and blood (**c**). The color usage represents the different subsets of T cells: Th17 (green), Th22 (blue), Th1 (purple), Th2 (yellow), and Th IL-10+ (pink). In the right side of the figure, relapse phase is depicted in two stages, early (4 weeks, towards the center of the figure), and late relapse (8 weeks, far right), symbolizing the different dynamics at different timepoints. For spleen and blood, the area below the dotted line represents outcomes that were below the baseline of results observed in uninfected animals. Image created with BioRender.com.

A comparative assessment demonstrates that the greatest changes in Th populations occur in the lung during ATB and in spleen at relapse (Fig. 6a, b). As visually displayed in Fig. 6a, ATB of the lung was generally characterized by expansion of heterogenous Th populations. Following drug treatment, these populations contracted at varying rates. Th17 and Th2 cells appeared to contract quickly, while Th22 and especially Th1 cells displayed a more protracted response. During relapse, both Th1 and Th17 populations increased again while Th22 cells were noticeably absent. An interesting observation was the consistent increase of Th17 cells in blood, spleen, and lung at 4 weeks (Fig. 6), suggesting potential for Th17 to serve as an early indicator of Mtb regrowth. These findings demonstrate that the pulmonary Th response observed at relapse is subdued compared to that of ATB. In addition, the population dynamics of the Th cells in the blood, and especially the spleen, frequently differ from those observed in the lung compartment (Fig. 7).

Use of AG in our model for 4 or 8 weeks did not significantly impact Th populations in the lung or blood in the absence of Mtb infection (Supplementary Fig. 1b, c). In the spleen, treatment with AG led to a moderate change in Th17 and Th22 percentages that were opposite in directionality from those observed in AG-mediated relapse of paucibacillary TB (Supplementary Fig. 1b). The Th1 population significantly increased in spleen due to 8 weeks of AG treatment (Supplementary Fig. 1c). However, the increase due to AG in uninfected controls was limited (0.4%) compared to the marked increase observed due to relapse (8% in Fig. 5b). In brief, use of AG has minimal effects on baseline Th populations.

## Discussion

The basis for development of post-drug TB relapse is poorly understood due to the lack of comparative research and relies heavily on paradigms for latent TB reactivation. Development of latency is associated with lower risk of TB upon re-infection and generation of central memory T cells including Th1 populations^39,40^. Progression from latent to active TB is classically understood to occur due to a new immune compromise such as HIV infection, diabetes, or other co-morbidity^41,42^ that drives regrowth of dormant organisms. Recent evidence also suggests potential for a spectrum of Mtb persistence in many individuals that includes metabolically active bacilli^19,43^. Investigations that inform our knowledge of immune responses in the post-drug period, including relapse, are limited to a few studies in murine models and observations of human plasma or blood populations.

As illustrated in Fig. 7, our findings demonstrate how drug treatment and regrowth after primary Mtb infection impact the Th cell populations that are potential targets for therapeutic vaccines or host directed therapies. Importantly, several established endpoints of Th-mediated immune response to ATB are reproduced as validation of the model for understanding the changing landscape after drug treatment and relapse. A relevant example is the expansion of Th1, Th17, and Th1Th17 cells observed in the lung during ATB compared to the blood, as previously described in human specimens^33,34^. Activation of a strong pro-inflammatory cytokine profile in the lung, including TNF, IL-6, IFN-γ, and IL-17, are also well described immune responses to primary Mtb infection^44^ that are reproduced in the model. Results from our analysis of an expanded repertoire of Th subpopulations and polyfunctionality also advances the understanding of the cellular immune responses in ATB. An especially noteworthy finding was that Th22, and Th cells that include IL-22 as part of the polyfunctional profile, are a prominent immune signature in the ATB stage.

The decrease in several Th populations which we observe in the PDTB stage is consistent with reduced innate immune activation as Mtb burden declines following antibiotic administration. An interesting observation was the enduring pulmonary Th1 response in the PDTB stage despite a decline of other Th populations. Accordingly, soluble IFN-γ and TNF remained elevated in the lung during PDTB although the overall levels were lower compared to ATB. These results suggest a reduced requirement for antigen or immune signaling needed to maintain the pool of Th1 effectors. The observed increase in Th2 and Th IL-10+ cells following antibiotic treatment, which occurred irrespective of infection status, is also notable. These subsets could be deleterious during TB, as Th2, T reg cells, and IL-10 have been shown to reduce protective central memory T cell responses to BCG vaccination and impair Mtb clearance^45–47^. Further, the predominance of these populations during the PDTB stage indicates a shift from a Th1 to a Th2 cytokine environment that is further supported by observations of increased IL-4, IL-13, and ratio of IL-10 to IFN-γ in the lung supernatants. Development of this immune regulatory environment could be a mechanism whereby suppression of T cell memory and effector function contributes to incomplete Mtb eradication.

Importantly, our results reveal that the Th response of TB relapse is distinct from ATB and is characterized by a diminished T cell response consisting predominantly of Th1 and Th17 cells. The generalized suppression of total, as well as specific, Th populations may reflect protracted effects of the immune regulatory environment observed in PDTB and immune exhaustion due to persisting antigen^48^. The predominance of Th1 and Th17 populations, and the early Th17 response observed at 4 weeks of relapse, could be due to timing of immune activation, differences in tissue resident pools, and balance of immune signals (e.g., cytokines, chemokines) as Mtb regrowth occurs. A lower bacterial burden that we and others observe during relapse compared to ATB^29,49^ may also contribute to this effect or reflect immune pressure mediated by Th1 and Th17 cells. Both populations have established immune roles in ATB and are key targets of TB vaccine efforts^50–52^. Th1 cells are expected to be protective during relapse due to the essential function of IFN-γ in immunity to Mtb^52,53^. On the other hand, past infection is also known to generate strong Th1 responses despite a lack of protection against a subsequent Mtb re-infection^54^. Our observations that Th1 cells and IFN-γ cytokine are the only responses observed at all stages (ATB, PDTB, and relapse) reinforce the concept that IFN-γ is not sufficient as a correlate of protection^55–57^. The Th17 signature we observe in the lung, spleen, and blood at 4 weeks suggest Th17 cells may serve an “early responder” role in relapse. The trend toward decline of Th17 cells at 8 weeks, along with evidence for similar bacterial burden, suggests that Th17 cells are also not sufficient as a correlate of protection.

The abundance of Th22 observed during ATB, and the notable lack of Th22 during relapse, may also identify a potentially important finding. IL-22 plays a regulatory role in host responses and was only recently shown to participate in anti-mycobacterial immunity^52,58,59^. Increased IL-22 cytokine is observed in bronchoalveolar lavage and extrapulmonary sites of individuals with active TB and has been shown to activate macrophage antimicrobial activity against Mtb infection^60–62^. Mice deficient in IL-22 are no more susceptible to primary infection with Mtb H37Rv, but greater mycobacterial growth was recently observed in animals challenged with the more virulent HN878 strain^63–65^. Furthermore, increased CD3 + IL-22+ cells have been described in lung and granulomas of rhesus macaques following Mtb infection^66^. Th22 cells were significantly increased during ATB and the largest polyfunctional group of Th cells during ATB were those producing both IFN-γ and IL-22. The notable lack of Th22 cells and IL-22 cytokine during relapse may identify an important defect in cellular immunity. This outcome may also indicate a broader suppression of IL-22 cytokine gene expression given that IL-22 is produced by diverse leukocyte populations in addition to Th cells.

Polyfunctional cytokine profiles (e.g., IFN-γ, TNF, IL-2) are frequently associated with improved outcomes in TB and other bacterial disease models, though to date, a profile that definitively correlates with protection against Mtb is lacking (for review see Lewinsohn, 2017^67^). Protection of non-human primates from Mtb challenge following intravenous BCG vaccination was recently shown to correlate with increases in total Th cells that express IL-2, IL-17, TNF, or especially IFN-γ, following in vitro antigen exposure^24^. In that study, differences in Th polyfunctionality were not observed between protective and non-protective immunization routes. We also observed that monofunctional Th cells producing IFN-γ were the most abundant population observed ex vivo, in all stages, and especially PDTB. Several new observations in the current study also warrant further assessment, including: (1) the increase in Th22 cells, and prevalence of IL-22 as part of polyfunctional profiles that included IL-17 and IFN-γ during ATB; (2) development of polyfunctional Th populations populated by IL-4 or IL-10 during PDTB; and (3) the dominant IL-17 signature of polyfunctional Th cells during relapse. The overall increase in polyfunctionality observed at relapse compared to ATB was surprising and suggest that T cells may be differentially activated at this stage.

The Th profile that we observed in different tissue compartments and stage of disease strongly suggest an important role for microenvironments and other immune factors. Importantly, the Th cells do not appear to simply reflect changes in bacterial burden. During ATB, Th1 increased in both the lung and blood proportional to the lung bacterial burden. In contrast, pulmonary Th17 increased during ATB in the absence of an expansion in blood. A similar dichotomy in the Th1 and Th17 bias in blood was previously described in human subjects with TB^68,69^. Following anti-tubercular treatment, we observed that Th1 cells returned to baseline in the blood but remained elevated in lung despite non-detectable mycobacteria and markedly reduced lung cytokines. Differences in splenic populations further supported microenvironment effects. Th1, Th2, Th22, and Th17 populations were more abundant in spleen during late relapse TB compared to ATB and also frequently differed from lung and blood at the same stage of experimental treatment. The greater abundance of Th cells in the spleen may be due to the efficiency of recruitment of memory and effector T cells populations to the spleen^70^. In addition, the retention of memory cells in secondary lymphoid organs along with restricted migration to organs such as the lung^71^ may be a consideration. Future studies of chemokine or adhesion marker changes could explain these variable responses in different organs. These differences also suggest limitations to extrapolation of results obtained from human blood when making determinations of immune status in lung or other organs of persons with TB.

Effects of anti-tubercular drugs on the gut microbiome may also contribute to the Th profile that we observed in the PDTB and TB relapse stages. Anti-tubercular treatment induces microbiome dysbiosis for up to 1.2 years after therapy completion^72,73^, and mice with altered microbiota are more prone to Mtb colonization^73,74^. People with a history of successful TB treatment have greater risk for re-infection than previously uninfected individuals^75^. Alterations in the gut microbiome can change the immune responses of the lung, suppressing IFN-γ and IL-17 expression and increasing regulatory Th populations^76,77^, similar to our observations. Loss of T-cell reactivity to Mtb epitopes has also been reported in persons who have completed TB chemotherapy; an effect postulated to involve epitope homologs of microbiome^78^. Understanding how anti-tubercular drugs change the microbiome and affect the balance of Th populations with protective and regulatory functions is thus important for development of vaccine strategies and adjunctive therapies. A greater understanding of the gut/lung axis may also identify opportunities to optimize Th bias through interventions that restore the gut health.

The changes in Th profiles that we describe may have important clinical implications once validated in human subjects completing TB treatment. People who develop relapse often report clinical symptoms similar to acute TB, but with decreased frequency. Chest pain, cough, nausea and vomiting are reported less often during relapse, compared to those with a presumptive new infection^79,80^. The decreased symptoms may correspond with a lower bacterial burden at relapse, as we and others observed in mouse models of relapse or reinfection^29,49,81^ although this has not been investigated in human lung. Other clinical findings at relapse, however, suggest development of a pro-inflammatory immune response or delayed resolution. Patients with TB relapse have been shown to present greater pulmonary cavitation, hemoptysis, fibrosis, and infiltration, among other abnormal findings^80^. Th17 have well described roles in inflammatory lung damage^82,83^ and are long-lived memory cells with a strong capacity for self-renewal^84,85^. Due to the importance of IL-22 in mucosal wound-healing^86,87^, especially in TB^88^, loss of Th22 cells following drug treatment may remove an important regulatory mechanism. The shift in Th cell bias away from Th22 and toward Th17 during relapse could thus be a mechanism of pathogenesis.

There are some caveats that should be addressed relevant to our observations. Employment of AG to accelerate relapse, via NOS2 inhibition and loss of immune pressure by nitric oxide (NO), has been previously described^32^. NOS2 is activated downstream of Th-1 derived cytokine (e.g., IFN-γ, TNF) signaling and imbalances in NOS2 and arginase metabolism of L-arginine can promote immune regulatory outcomes such as increased regulatory T cells^89–92^. NOS2-deficient mice infected with Mtb, however, have been shown to have similar IFN-γ and increased pro-inflammatory cytokines (e.g., IL-6, and IL-1β) in lung compared to wild type^93^. Our results showed that AG use had minimal effects on baseline Th profiles and number of Th cells was only moderately reduced compared to ATB. The observed lung cytokine profile also was not suggestive of polarization toward a regulatory microenvironment. IFN-γ was the most prevalent lung cytokine, Th1 and Th17 subpopulations predominated, and Th IL-10+cells did not expand. Nonetheless, the potential for AG to affect Th outcomes through L-arginine supply or other factors cannot be excluded. Targeted approaches that reproduce clinically important causes of relapse, instead of NOS2 inhibition or broadly suppressive alternatives such as dexamethasone, would further expand our understanding of the immunological basis of relapse.

Use of anti-CD3 and anti-CD28 to assess ex vivo Th populations is advantageous because the total Th population can be observed in the absence of epitope bias^94^ and relevant inferences made based on experimental inputs (Mtb infection and drug treatment) and controls. Many Mtb T cell epitopes are well conserved^95,96^, however, expression can vary in the course of TB disease and the full repertoire is unknown^97^. Long term, approaches that employ tetramers displaying MHC class II epitopes in combination with T cell memory phenotype markers^98^ will be important for efforts to understand effects on specific T cell memory compartments. Mouse strain and Mtb lineage may also play a role in the observed immune responses. Balb/c mice produce greater increases in IFN-γ and IL-17 due to BCG vaccination when compared to C57BL/6^99^. The HN878 strain used in our experiments is associated with increased rates of relapse in human subjects and a mouse model^49,100–103^ and activates greater Th17 and Th22 responses compared to H37Rv^63,104^. These factors are important to consider in comparative models and may also have clinical relevance since host immune status varies and HN878 strain is widely distributed in the world^100^.

In conclusion, we identified dynamic shifts in Th populations between active and relapse TB that demonstrates a need to define subset-specific roles in disparate stages of disease. The early response of Th17 cells, and the failure of Th22 cell re-expansion during relapse that we observed, especially warrant further exploration. Our findings may additionally have relevance to understanding immune outcomes that permit establishment of Mtb re-infection, although comparative studies will be needed. Host directed interventions that promote Th17 effector function during drug treatment may promote earlier Mtb clearance, allowing for a shortened antibiotic regimen and reduced risk for relapse. The development of a regulatory microenvironment (e.g., IL-10 producing Th cells) that we observe following drug treatment may also represent a previously unknown challenge for implementation of vaccines intended as adjunctive therapy or preventive applications at this stage of treatment. After drug treatment, a bias toward Th1 and Th17, and away from Th22 and Th IL-10+, could promote tissue damage that favors Mtb replication. Determining the role of cytokine bias and/or T cell anergy as factors that limit anamnestic Th response to Mtb regrowth are thus important areas of investigation. Therapeutic vaccines that target Th22 cells may be an important avenue for promoting immunity in adjunctive or preventive treatment. Clinical approaches (e.g., gut microbiome support) which restore the balance of cytokines that determine Th bias may be avenues to improve host immunity and vaccine efficacy. Future investigations should also define the memory phenotype and epitope restriction of the Th subpopulation landscape across disease and treatment. Long term, these advances will fill an important knowledge gap that currently limits rational design of vaccines to prevent TB relapse.

## Methods

### Ethics statement

All animal experiments complied with and were approved by the University of Texas Medical Branch (UTMB) Institutional Animal Care and Use Committee under protocol 1501001B.

### Bacterial culture

Mtb strain HN878 (Beijing lineage) was cultured using Middlebrook 7H9 broth (Difco Laboratories, Sparks, MD, USA), supplemented with 0.5% (v/v) glycerol, 0.05% (v/v) Tween 80, and 10% oleic acid-albumin-dextrose-catalase enrichment (OADC, Becton, Dickinson and company, Sparks, MD, USA). Mtb was propagated at 37 °C until reaching an OD_600_ of 0.5 and diluted to 3 × 10^5^/ml of 7H9 growth media for aerosol delivery of 100 CFU/ mouse.

### Experimental mouse model

All animal and bacterial experiments were performed in Biosafety level 3 (BSL3) laboratory and animal facilities approved by the Centers for Disease Control and Prevention. Experiments were performed in accordance with guidelines and protocols approved by the UTMB Department of Biosafety. Seven-week-old, female, C57BL/6J mice, supplied by The Jackson Laboratory were used. Mice were infected via aerosol inhalation exposure in the Animal BSL3 (ABSL3) Aerobiology procedure suite, by using a Biaera AeroMP system with a Collison nebulizer with 6-jet nozzle, for 7.5 min^98^. To assess immune differences during ATB, PDTB, and relapse TB, we reproduced a model of TB relapse that employs inducible nitric oxide synthetase (iNOS or NOS2) inhibition by AG to promote regrowth of paucibacillary Mtb infection following non-sterilizing drug therapy, using methodology previously described by Botha and Ryffel^31^. One variation to the protocol was use of an infectious dose of 100 CFU instead of 30 CFU. Briefly, after 4 weeks of Mtb infection, oral anti-tubercular antibiotic treatment with RIF and INH was administered to both infected and uninfected mice through ad libitum provision of 0.1 mg/mL each drug in sterile drinking water. Water bottles were wrapped in aluminum foil to reduce degradation of drugs from light and changed twice per week.

Mice received antibiotics from week 4 to 12 post-infection with Mtb. From week 15 to 23, mice received water with 2.5% w/v AG(NOS2 inhibitor) to induce relapse and supplemented with 10% glucose w/v to improve palatability. The water was changed once per week, and was filter sterilized before being given to mice for ad libitum consumption. Mice were anesthetized under 3% isoflurane, and humanely euthanized with anesthesia overdose, followed by cervical dislocation at end of ATB (4 weeks), end of anti-tubercular treatment (PDTB, 12 weeks), and after 4 or 8 weeks of relapse (week 19 or 23) (Fig. 1a). Blood and organs (lung, liver, and spleen) were collected and used for microbiological and immunological assessments.

### Determination of viable Mtb in tissues

Lung (superior, middle, inferior and post-caval lobes), or liver (left superior lobe) were collected in 1 mL of sterile PBS and disrupted using a tissue grinder (Covidien). Viable Mtb in tissue homogenates were enumerated by serial dilution in sterile PBS, and growth on 7H11 agar plates as described previously^30^. Plates were incubated for up to 4 weeks at 37 °C prior to CFU quantification. The remaining supernatants from disrupted lung were harvested after centrifugation at 1500 × *g* for 10 min, and frozen at −80 °C for subsequent analysis.

### Assessment of cell populations and polyfunctionality

Left lung, spleen, or intracardiac blood were collected for flow cytometric analysis of leukocyte populations. Blood was collected in heparin coated tubes, and tissues were collected in cold RPMI media (Gibco, Cat. 11875-093), supplemented with 10% FBS, 1% penicillin/streptomycin, 1 mM sodium pyruvate, 10 mM HEPES, 2mM L-glutamine, and 1% MEM non-essential amino acids (complete RPMI, or cRPMI). Single cell suspensions of lung tissue were collected following mechanical and enzymatical disruption in the presence of Collagenase IV (0.7 mg/mL) and DNase I (30 µg/mL) for 45 min. Enzymes were inactivated with 1 mL of FBS/sample and tissues were further disrupted by pressing fragments through a 70 µM cell strainer. Larger cells (e.g., fibroblasts) and debris were removed by low speed (60 × *g*/1 min) centrifugation followed by isolation of leukocytes and other smaller cells in the supernatant by centrifugation at 350 × *g* for 5 min and resuspension in cRPMI. Splenic cells were isolated by passing tissue through a 100 µM cell strainer and washing with cRPMI. Isolated lung, spleen, or blood cells were activated for 5 h at 37 °C with plate bound anti-CD3 antibody, and addition of anti-CD28 antibody (1 µg/mL each, Biolegend #100302 and 102102, respectively) in the presence of GolgiStop (BD) to retain intracellular cytokines. Viability was assessed using a fixable live/dead marker (live/dead NIR, Thermo Fisher Scientific #L10119) followed by incubation with antibodies to CD3- PE-Vio770 (1 µL, Miltenyi Biotec #130-116-530) and CD4-BUV395 (1 µL, BD Biosciences #563790). An RBC lysis of blood samples was performed using BD FACS Lysing solution prior to fixation as part of the intracellular staining protocol. Fixation and permeabilization for intracellular staining were performed by using Cytofix/Cytoperm (BD) fixation, with Perm/Wash permeabilization followed by incubation with antibodies to intracellular molecules of interest: IFN-γ-BV510 (3 µL, BioLegend #505842), IL-10-PerCP-Cy5.5 (1 µL, BioLegend #505028), IL-17A-BV711 (3 µL, BioLegend #506941), IL-22- PE (1 µL, BioLegend #516404), and IL-4-BV786 (1 µL, BD Biosciences #564006). Inactivation of sample infectivity was performed with 4% ultrapure formaldehyde (Polysciences) for 48 h and followed by acquisition on a LSR II (Fortessa) flow cytometer. Subsequent analysis was performed using FCS Express 6 software (de Novo, Inc). Polyfunctionality analysis of the viable lymphocyte gate identified with FCS Express was further assessed by combinatorial cytokine data selected by Boolean gating and subsequent analysis of Th cells by coupled PESTLE (http://www.drmr.com/pestle.zip) and SPICE software^105^.

### Cytokine assessment via multiplex ELISA

Frozen plasma and supernatants from lung and spleen were exposed to 5 MRAD of γ-irradiation on dry ice to permit safe use and pathogen inactivation was confirmed by lack of Mtb growth on 7H11 agar plates as described^106^. Cytokines were measured by using the Legendplex multi-analyte flow assay kit mouse Th cytokine panel (No. 740741, Biolegend), followed by acquisition on a LSR II (Fortessa) flow cytometer and analysis using the Biolegend online data analysis software.

### Statistical analysis

All graphs and statistical analysis were generated with GraphPad Prism 8, and data is shown as mean ± SEM. A two-tailed unpaired Student’s T-test was used to assess differences in comparisons of two intra-stage experimental groups (ATB or PDTB). A one-way ANOVA with Tukey’s multiple comparison test was used to compare the relapse phases, and for comparison of stages across time. Statistical relationships between continuous variables were determined using a Pearson’s Correlation Coefficient and true outliers were identified by the ROUT method. Significance was considered with any *p* value < 0.05, and a trend in significance was considered where *p* < 0.1.

### Reporting summary

Further information on research design is available in the Nature Research Reporting Summary linked to this article.

### Supplementary information

Supplementary Figures

REPORTING SUMMARY

## Data Availability

Authors can confirm that all relevant data are included in the paper and/or its supplementary information files.
