# Supplementary material for: Helper T cell bias following tuberculosis chemotherapy identifies opportunities for therapeutic vaccination to prevent relapse
Source: NPJ Vaccines. 2023 Oct 28;8:165. doi: 10.1038/s41541-023-00761-4 (PMC10613213; doi:10.1038/s41541-023-00761-4)
Supplement: Supplementary file 1 — Supplementary Figures [file 41541_2023_761_MOESM1_ESM.pdf]

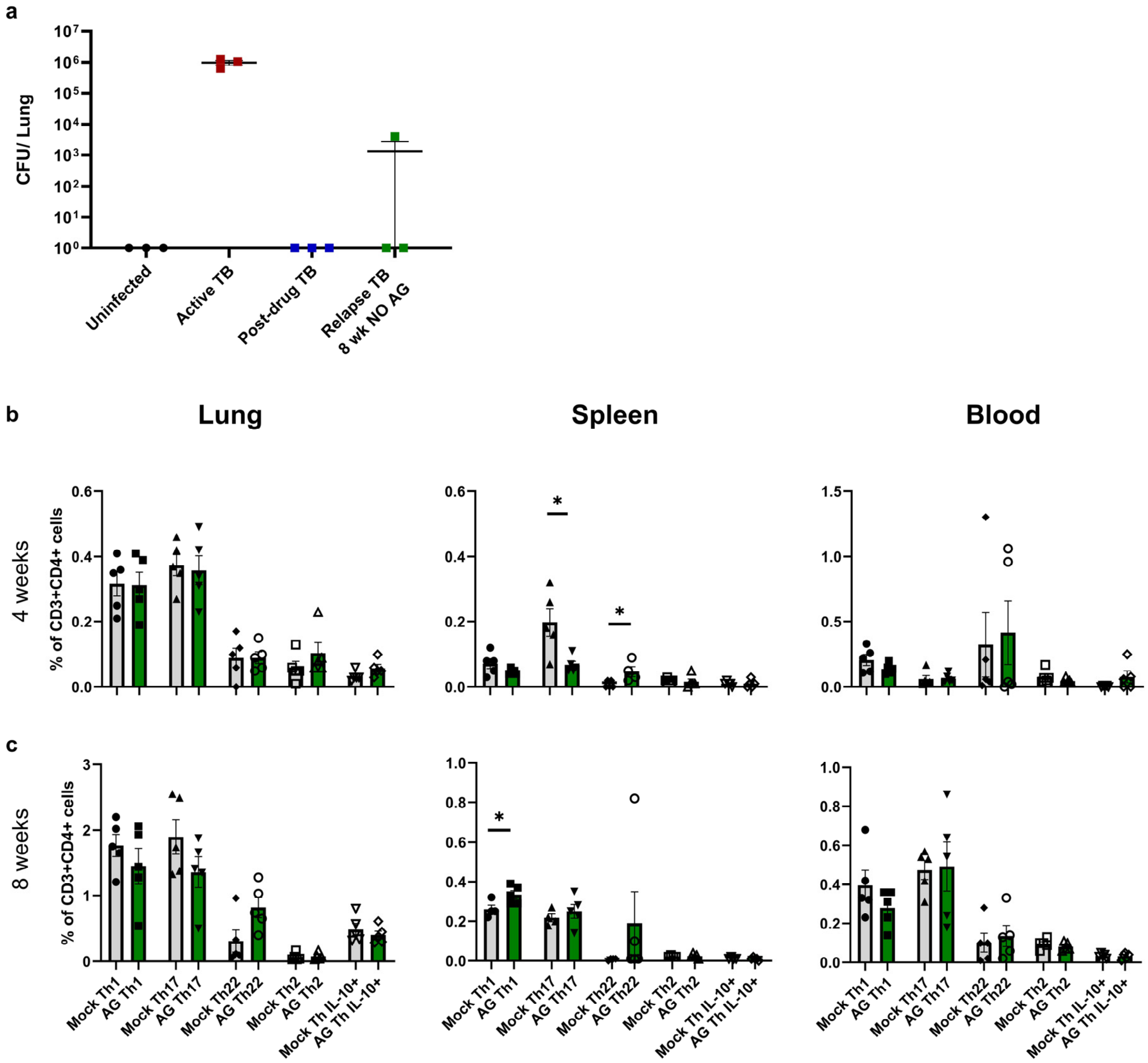

Supplementary Figure 1. Effect of aminoguanidine on relapse and on baseline Th cell responses. (a) Bacterial burden in lung across tuberculosis, post-drug treatment reaching paucibacillary state, and 8 weeks of relapse (n= 3 per group). Results show that only one of three mice relapsed in absence of aminoguanidine (AG). (b-c) Effect of AG on percentage change of T cell populations after 4 weeks (b) or 8 weeks (c) of AG administration (n=5 per group). Uninfected mice were given 2.5% AG + 10% glucose (for palatability) in water *ad libitum*, for 4 or 8 weeks. T cell population percentages among CD3+CD4+ cells in the lung (left), spleen (center) and blood (right) were analyzed by flow cytometry. Data are shown as mean ± SEM. *p<0.05 (Student's T-test).

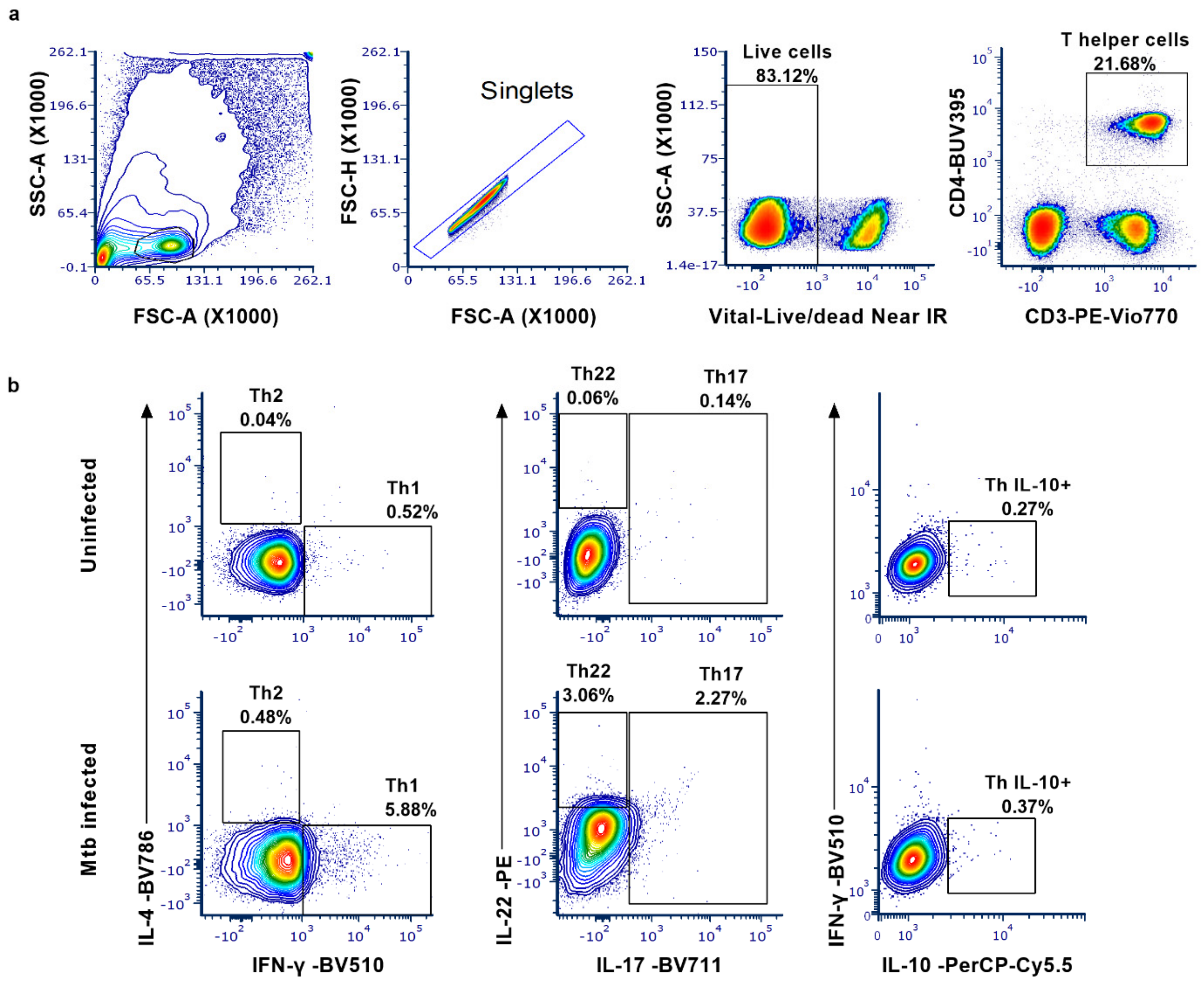

Supplementary Figure 2. Flow cytometry gating strategy and representative T helper subset analysis display. Isolated lung cells were acquired after disaggregation, activation with anti-CD3/anti-CD28/Golgi Stop, and detection of extracellular and intracellular markers. (a) Lymphocytes were selected based on forward (FSC-A) and side scatter (SSC-A) characteristics. Single cells were further selected by exclusion of doublets using FSC-A and FSC-H gating and viability determined based on exclusion of vital stain. Expression of cytokines by T helper cells was determined based on detection of surface CD3 and CD4 markers and (b) intracellular cytokines to enable determinations of Th subsets as follows: Th1 (IFN-γ+IL-4-), Th2 (IL-4+ IFN-γ-), Th17 (IL-17+), Th22 (IL-22+IL-17-) and Th IL-10+ (IL-10+ IFN-γ-). Examples of cytokine expression in uninfected (upper panels) and Mtb-infected (lower panels) samples are shown.

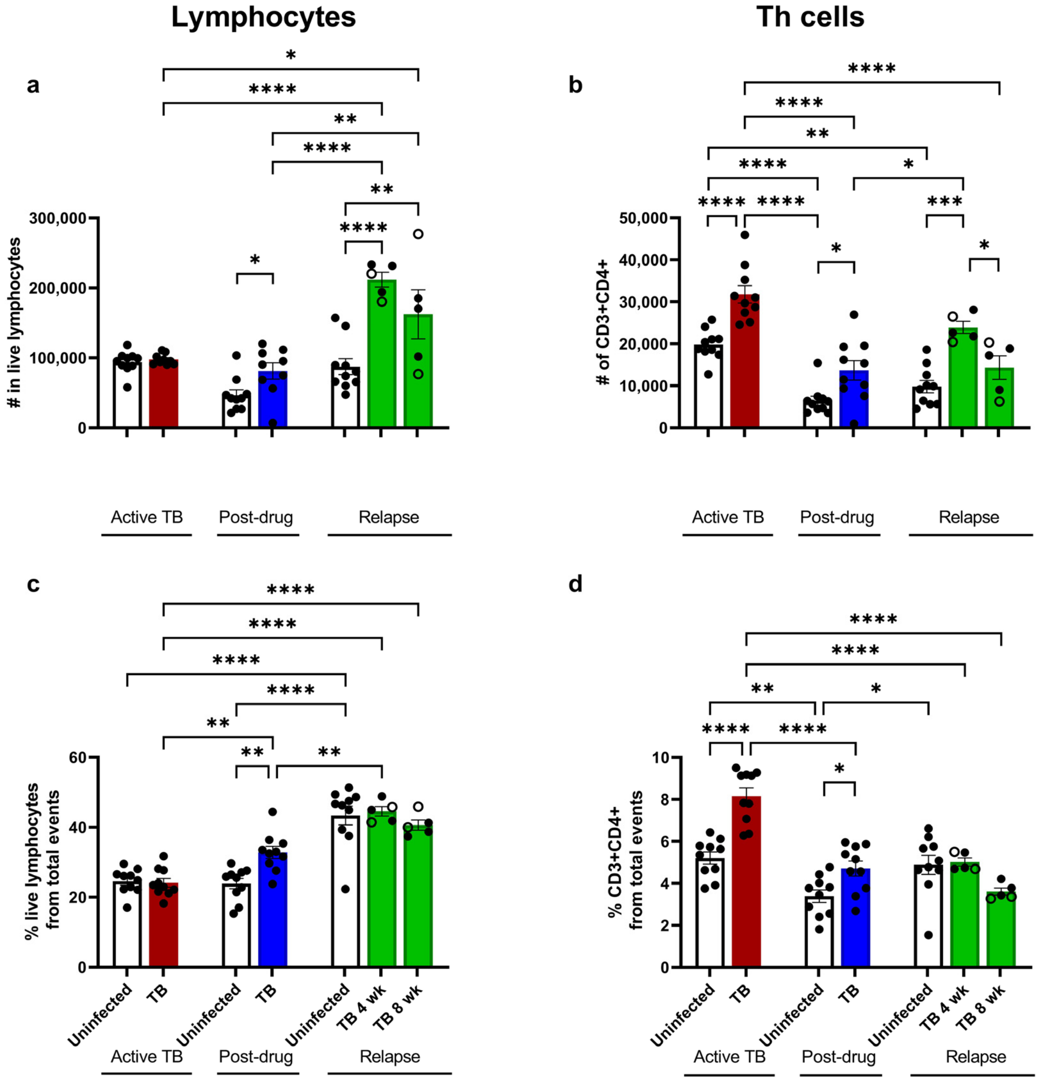

Supplementary Figure 3. Total lymphocyte and Th cells at different disease stages. Percentage and number of lymphocytes and T cells in lung after active TB (red, n=10), post-drug treatment (blue, n= 10), and 4 or 8 weeks post bacterial regrowth (green, n= 5/ group), compared to the uninfected controls (white, n= 10/ group). (a) Number of live lymphocytes at each phase acquired from the total half lung acquisition. (b) Number of T cells (CD3+CD4+, live lymphocyte size) acquired from the total half lung. (c) Percentage of live lymphocytes among total events from the lung. (d) Percentage of T cells among the total events. Open green symbols indicate animals that did not relapse. A Student's t-test was used to determine differences between two groups within treatment phase. Comparisons at relapse, or across treatment stages were analyzed using one-way ANOVA followed by Tukey test for multiple comparisons. Data shown as mean ± SEM. *$p<0.05$, **$p<0.01$, ***$p<0.001$, ****$p<0.0001$

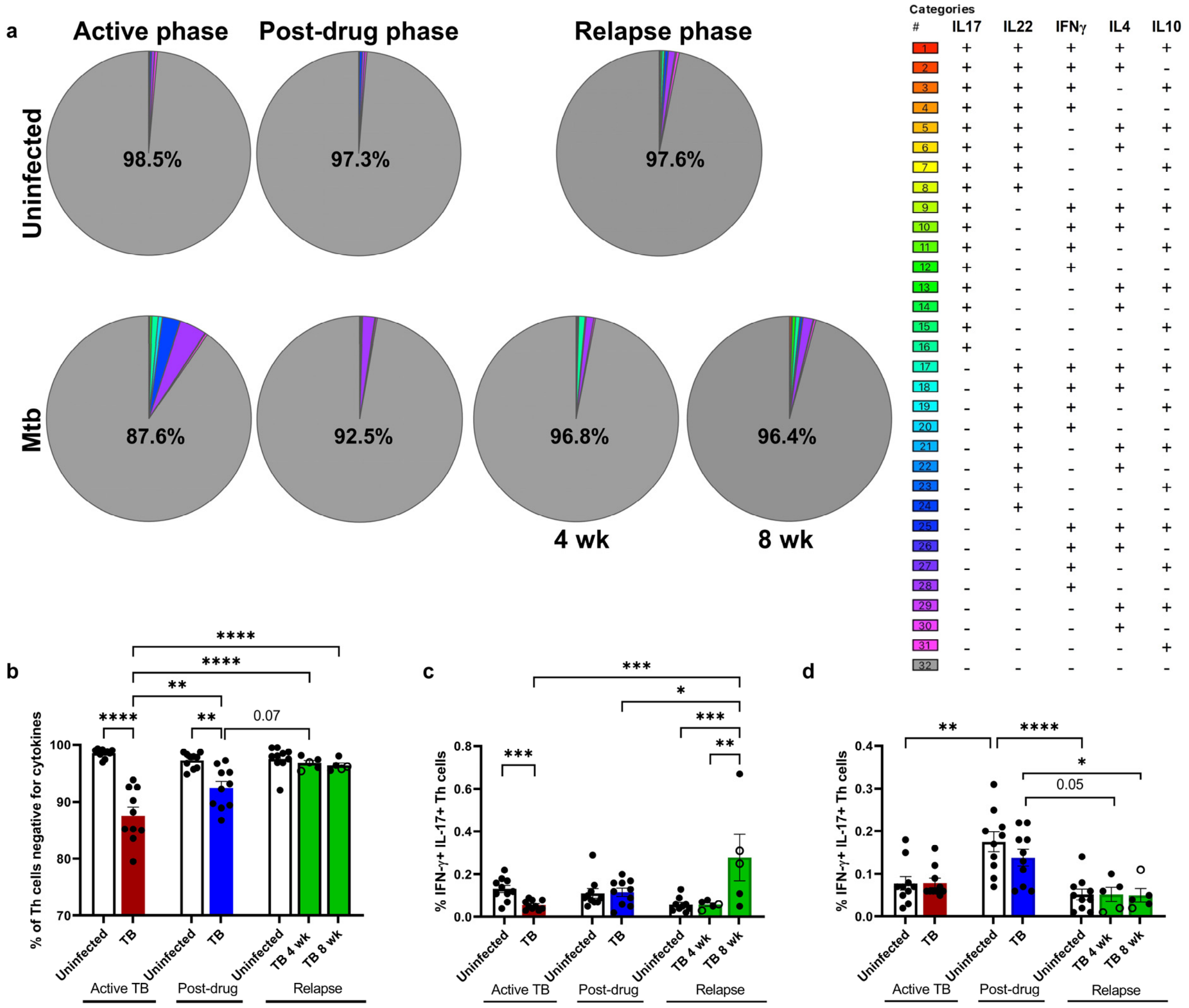

Supplementary Figure 4. Th cell polyfunctionality in lung, spleen, and blood. (a) Polyfunctional analysis of all CD3+CD4+ cells by Pestle and SPICE, across active (n= 10), post-drug (n= 10), and relapse tuberculosis phases (4 and 8 weeks, n= 5/ group), compared to the uninfected controls (n= 10/ group). In the pie charts, colors are depicted at the right. Percentages in the gray pie chart are the percentages of cytokine inactive T cells (group 32, not producing any of the 5 studied cytokines). (b) Percentage of inactive T helper cells in lung compared between groups. (c-d) Percentage of Th1Th17 cells (IFN-γ+IL-17+CD3+CD4+) among T helper cells, across the experimental phases, in spleen (c), and blood (d). Open green symbols indicate animals that did not relapse. Data shown as mean ± SEM. *p<0.05, **p<0.01, ***p<0.001, ****p<0.0001 (Student's T-test for active TB and post-drug phases, One-Way ANOVA followed by Tukey test for relapse phase, and comparison of phases across time).
